# The prevalence of awake bruxism and sleep bruxism in the Dutch adult population

**DOI:** 10.1111/joor.12787

**Published:** 2019-04-02

**Authors:** Peter Wetselaar, Erik (J. H.) Vermaire, Frank Lobbezoo, Annemarie A. Schuller

**Affiliations:** ^1^ Department of Orofacial Pain and Dysfunction, Academic Centre for Dentistry Amsterdam (ACTA) University of Amsterdam and Vrije Universiteit Amsterdam Amsterdam The Netherlands; ^2^ TNO Child Health Leiden The Netherlands; ^3^ Center for Dentistry and Oral Hygiene University Medical Center Groningen Groningen The Netherlands

**Keywords:** adult population, awake bruxism, bruxism, gender, prevalence, sleep bruxism

## Abstract

**Background:**

Awake bruxism and sleep bruxism are common conditions amongst adult populations, although prevalence data are scarce.

**Objective:**

This study aimed to assess the prevalence of awake bruxism and sleep bruxism in the Dutch adult population.

**Methods:**

As part of a large epidemiologic survey on oral health of the general Dutch adult population, a total of 1209 subjects were asked about their bruxism behaviour during the day and during their sleep. The collected data were subjected to stratified analysis by five age groups (25‐34, 35‐44, 45‐54, 55‐64 and 65‐74 years), socioeconomic status, and gender.

**Results:**

A prevalence of 5.0% of the total population was found for awake bruxism and of 16.5% for sleep bruxism. Regarding the five age groups, prevalence of 6.5%, 7.8%, 4.0%, 3.2% and 3.0%, respectively, were found for awake bruxism, and of 20.0%, 21.0%, 16.5%, 14.5% and 8.3%, respectively, for sleep bruxism. Women reported both awake bruxism and sleep bruxism more often than men. These differences were statistically significant. Concerning socioeconomic status (SES), both awake bruxism and sleep bruxism were more often found in high SES groups, being statistically significant for awake bruxism only.

**Conclusion:**

Sleep bruxism is a common condition in the Dutch adult population, while awake bruxism is rarer.

## 1 INTRODUCTION

In 2013, a group of experts defined bruxism as a repetitive jaw‐muscle activity characterised by clenching or grinding of the teeth and/or by bracing or thrusting of the mandible.^1^ It was stated that bruxism has two distinct circadian manifestations: it can occur during wakefulness (indicated as awake bruxism) or during sleep (indicated as sleep bruxism). Recently, a revision of the definition was made, in which awake bruxism (AB) and sleep bruxism (SB) are considered to be different behaviours observed during wakefulness and during sleep.^2^ Bruxism should not be considered as a disorder in otherwise healthy individuals, but rather as a behaviour, a physiological phenomenon, that can be a risk (and/or protective) factor for certain clinical consequences. Bruxism may be considered as pathological when a person experiences possible negative consequence, like pain in the masticatory system.^2^ Additionally, a diagnostic grading system was proposed of “possible,” “probable” and “definite” awake bruxism or sleep bruxism. Possible awake bruxism or sleep bruxism is based on a positive self‐report only, while probable awake bruxism or sleep bruxism is based on a positive clinical inspection, with or without a positive self‐report. Definite awake bruxism is based on a positive instrumental assessment, with or without a positive self‐report and/or a positive clinical inspection.^2^

A review regarding prevalence data amongst adult populations shows that studies on this topic are scare and have a wide variety.^3^ For this variety, several factors play a role, namely the fact that some researchers did not make the distinction between awake bruxism and sleep bruxism, nor the distinction between “possible,” “probable” and “definite” awake or sleep bruxism. For the assessment of “possible” awake or sleep bruxism, no consensus is reached on which questions and/or questionnaires should be used to set the diagnosis. Nevertheless, chairside questions and/or questionnaires are tools that can be applied relatively easy to larger groups of individuals. More studies are performed regarding sleep bruxism than awake bruxism. Studies using questionnaires resulted in varying prevalence data regarding sleep bruxism. Prevalence data were reported of 10.0% in 1106 Swedish adults,^4^ of 15.3% in 975 Danish adults,^5^ of 8.0% in 2019 Canadian adults,^6^ of 8.2% in 13 057 adults in three European countries (Germany, Italy and the United Kingdom with 4115, 3970, and 4972 subjects, respectively),^7^ of 14.0% in Israelian adults^8^ and of 8.6% in 6357 Canadian adults^9^ (see Tables 4 and 5).

Regarding the assessment of awake bruxism, to our knowledge, three studies are published, using questionnaires, so to set a “possible” awake bruxism diagnosis. These studies were also mentioned above, since these authors report on both sleep bruxism and awake bruxism. The Swedish study reported prevalence data of “possible” awake bruxism of 20.0%,^4^ the Danish study of 22.1%^5^ and the Israelian study of 31.0%^8^ (see Table 5).

Regarding the assessment of the “probable” diagnosis of sleep or awake bruxism, also here no consensus is reached. With regard to the clinical examination, several signs and symptoms are reported to be associated with sleep and awake bruxism. Signs are extra‐oral observable muscle hypertrophy, intra‐oral signs in the soft tissues (as, eg, indentations in cheek, tongue and/or lip),^10^ in the dental hard tissues (as, eg, intrinsic mechanical tooth wear, crumbling teeth, cracked teeth or tooth mobility),^10^, ^11^ and (frequent) failures of restorations (direct and/or indirect) or failures of (supra‐structures on) implants.^12^, ^13^ Described symptoms are TMD pain (arthralgia, myalgia, headache attributed to TMD), and stiffness or fatigue in the masticatory muscles.^10^ To our knowledge, a comprehensive clinical examination to detect awake bruxism or sleep bruxism was not used in any prevalence study in adults so far.

Regarding the assessment of the “definite” diagnosis of sleep bruxism, recently a study was published by Maluly and coworkers.^14^ They used both a polysomnographic recording and a questionnaire to asses sleep bruxism in a representative sample of 1042 Brazilian adults. The results indicated that the prevalence of sleep bruxism, indicated by questionnaires and confirmed by PSG, was 5.5%; with PSG used exclusively as the criterion for diagnosis, the prevalence was 7.4% regardless of sleep bruxism self‐reported complaints; with questionnaires alone, the prevalence was 12.5%.^14^ To our knowledge, no study regarding the “definite” diagnosis of awake bruxism has been performed.

The aim of this study was to assess the prevalence of awake bruxism and sleep bruxism in the Dutch adult population in different age groups, for both genders and for different socioeconomic status. Because this research was part of a comprehensive investigation of the oral health of the general Dutch adult population in 2013, it was only possible to assess awake bruxism and sleep bruxism with the use of chairside history taking, leading to the diagnosis “possible” awake bruxism and “possible” sleep bruxism.

## 2 MATERIALS AND METHODS

### 2.1 Study sample and recruitment

Data were collected from April 2013 to November 2013 as part of a large epidemiologic survey of oral health and preventive behaviour amongst Dutch adults.^15^ The survey was performed in ‘s‐Hertogenbosch, a medium‐sized city in the southern part of The Netherlands that can be considered to be representative of the general Dutch population in terms of sociodemographic indicators.^15^, ^16^ Health Insurance companies were asked (under the authority of the National Health Care Institute (Zorginstituut Nederland, ZINL) to provide the names and addresses of their clients aged 25‐75. A total of 87 075 names and addresses were provided. A stratified sample of 6904 people (including edentulous individuals) was selected.

All eligible individuals were invited to participate in this study and visit a mobile dental examination facility temporarily located in their city of residence. Eighty‐two per cent of those invited to participate (5661 individuals) stated that they were not interested in participation (51% of this group were male, 36% had higher education, 77% indicated that they did not have enough time or interest to participate, and 10% declined because of dental anxiety). A total of 1755 adults (56% of which were female) were included in the epidemiologic study. For the purpose of this study, all edentulous participants were excluded from the analyses so only data of the dentate participants were analysed (n = 1209).

This study was judged by the Central Committee on Research Involving Human Subjects (CCMO) not to fall under the provisions of the Medical Research Involving Human Subjects Act. It was furthermore decided that the study met all requirements of the Personal Data Protection Act (Approval No. m1501261).

### 2.2 Procedure

All participants filled in a questionnaire giving details of their sociodemographic and dental status, and their dietary and oral hygiene behaviour. Before they underwent an oral health assessment in a dental chair, the questions regarding awake bruxism and sleep bruxism were asked. The interviews were performed by experienced and calibrated dentists. Socioeconomic status was defined by the level of education. Level of education was divided into low and high, based upon the intellectual challenges offered by the Dutch education system. High education was defined as higher general secondary education or higher. All other education was defined as low education.

### 2.3 Questioning

For the purpose of this study, the following two questions were asked, modified from van der Meulen and coauthors:^17^ (a) do you grind your teeth or do you clench your jaws during wakefulness? and (b) did someone notice or are you aware yourself that you grind your teeth or clench your jaws during sleep? Regarding these questions, the participants could only answer yes/no/I don't know; additional questions regarding frequency and timeframe were not asked.

### 2.4 Statistical analysis

Participants were stratified in five age groups (25‐34, 35‐44, 45‐54, 55‐64 and 65‐74 years), gender and socioeconomic status (SES). Based on the power calculations, a total minimum of participants in each age group was calculated. As soon as this required number was reached, further efforts to include participants of that age were ceased. For every age group, this minimum number of participants was reached.

To identify whether the people that declined to participate differed from the participants, a modest non‐respondents survey was executed. This included amongst others age, gender and SES. It was found that this differed somewhat in SES. Because of that reason, the results were weighed by the factors that were calculated by Statistics Netherlands.

Kruskal‐Wallis tests and chi‐square tests were used to identify possible differences between age groups, gender and SES. All analyses were performed using IBM SPSS Statistics 22.0 software (IBM Corp., Armonk, NY, USA).

## 3 RESULTS

### 3.1 Response rates

The response rate in this study was 17.5%. Of the total sample, female respondents made up 55.5% of the study population. Of all participants, 44.7% had a low SES.

### 3.2 Age

Table 1 shows the prevalence of awake bruxism and sleep bruxism stratified in age groups of 10 years (25‐34, 35‐44, 45‐54, 55‐65 and 65‐74). Participants in the two youngest age groups reported more frequently to have this condition (6.5% and 7.8%) than the three older age groups. This difference is marginally statistically significant (*P* = 0.05). Considering sleep bruxism, higher prevalence rates were reported than for awake bruxism, with the two youngest age groups showing the highest rates (*P* < 0.01).

**Table 1:** Prevalence of self‐reported awake bruxism and sleep bruxism divided by age groups

Age	n	No	% Yes	χ^2^	*P*
Awake bruxism					
25‐34	230	215	6.5	9.282	0.054
35‐44	257	237	7.8		
45‐54	272	261	4.0		
55‐64	283	274	3.2		
65‐74	167	162	3.0		
Total	1209	1149	5.0		
Sleep bruxism					
25‐34	230	184	20.0	14.628	0.006
35‐44	257	203	21.0		
45‐54	272	227	16.5		
55‐64	283	242	14.5		
65‐74	167	153	8.3		
Total	1209	1009	16.5		

### 3.3 Gender

Both awake bruxism and sleep bruxism were reported more frequently by women than by men: 6.4% vs 3.2% for awake bruxism and 18.6% vs 13.9% for sleep bruxism (see Table 2). This difference is statistically significant for awake bruxism only (*P* = 0.05).

**Table 2:** Prevalence of self‐reported awake bruxism and sleep bruxism divided by gender

Gender	n	% Yes	χ^2^	*P*
Awake bruxism				
Male	538	3.2	6.681	0.010
Female	671	6.4		
Sleep bruxism				
Male	538	13.9	4.754	0.029
Female	671	18.6		

### 3.4 Socioeconomic status

Awake bruxism and sleep bruxism were reported more frequently by participant with high SES than by those with low SES: 6.3% vs 3.6% for awake bruxism and 16.8% vs 16.2% for sleep bruxism (see Table 3). This difference is statistically significant for awake bruxism and sleep bruxism (*P* = 0.05).

**Table 3:** Prevalence of self‐reported awake bruxism and sleep bruxism divided by SES

SES	n	% Yes	χ^2^	*P*
Awake bruxism				
Low	505	3.6	4.199	0.040
High	624	6.3		
Sleep bruxism				
Low	505	16.2	0.070	0.791
High	624	16.8		

## 4 DISCUSSION

This study aimed to assess the prevalence of awake bruxism and sleep bruxism in the Dutch adult population. Sleep bruxism is a common condition in the Dutch adult population, while awake bruxism is rarer. We will discuss about the results in the following sequence: (a) bruxism, awake bruxism and sleep bruxism; (b) possible, probable and definite bruxism; (c) the exact question regarding awake bruxism and sleep bruxism; (d) age patterns; (e) gender; (f) SES; (g) sample and sample size; and (h) future perspectives.

### 4.1 Bruxism, awake bruxism, sleep bruxism

Awake bruxism (AB) and sleep bruxism (SB) are considered to be two different behaviours observed during wakefulness and during sleep.^2^ Of course, this concept change is of influence how we look back to previous research, and how we will perform research in the future, meaning not assess bruxism, but assess awake bruxism and sleep bruxism separately. The strength of this study was that we assessed awake bruxism and sleep bruxism separately. Moreover, it is of importance to collect as much as prevalence data as possible, in order to broaden the available information of the “normality” that is bruxism considered now.

### 4.2 Possible, probable, definite

As mentioned in the introduction, to our knowledge, no epidemiological studies are performed in which a comprehensive clinical diagnosis was performed, meaning leading to the diagnosis “probable” awake bruxism or “probable” sleep bruxism. Important issue is that there is no agreement which signs and symptoms should be included in a comprehensive clinical assessment, consensus towards this is a necessity to compare future research. One can reason that so far, research is lacking due to the fact that performing such a prevalence study with a large sample size is too time‐consuming and therefore not executable. The same is applied regarding the assessment of the “definite” diagnosis of sleep bruxism, although recently a study was published by Maluly et al.^14^ This study is until now, the only one using polysomnographic recordings on such a large scale. Of course, this kind of research is of great importance, but difficult to implement because of the high costs. Furthermore, it is important to realise that a polysomnographic recording of only one night has to be considered with caution, since it is known that there is a variation between different nights.^18^ Moreover, using certain cut‐off criteria to determine the diagnosis “definite” sleep bruxism is on debate.^2^ Therefore, the strength of this study was that we assessed “possible” awake bruxism and ‘’ sleep bruxism in order to collect more prevalence data.

### 4.3 Exact question

For epidemiological studies on a large scale, interviewing persons by questionnaires or during an oral history remains the most easily accessible. This is the reasons why such studies are performed in the past^4^, ^5^, ^6^, ^7^, ^8^, ^9^ and remain necessary in the future. Shortcoming at this moment, is the fact that there is no consensus about which question(s) need to be asked, nor for awake bruxism nor for sleep bruxism. In the above‐mentioned epidemiological studies, different questions were used regarding sleep bruxism, as we did as well. Not only the used questions itself are different, also answer possibilities (yes, no,) or different time intervals (rarely, occasionally, often, very often, never, times per night, times per day, times per month) were asked for.^4^, ^5^, ^6^, ^7^, ^8^, ^9^, ^19^ In our study, we used the following question: did someone notice or are you aware yourself that you grind your teeth or clench your jaws during sleep? (yes/no). Remarkable is that the question itself seems more or less equal, but the difference is in the frequency‐part of the questions. That the question itself can influence the results remarkably, was shown by Khoury and coauthors, who reported an overall prevalence of 8.6% when asking regularly behaviour and a prevalence of 22.3% when asking regularly and on occasion behaviour together. Regarding the questions used for assessing awake bruxism, the same fluctuations in questioning were found as mentioned above regarding sleep bruxism.^4^, ^5^, ^8^, ^19^ In our study, we used the following question: do you grind your teeth or do you clench your jaws during wakefulness? These differences make comparison difficult and conclusions must be drawn with caution. Regarding the questioning, both for awake bruxism and sleep bruxism, consensus is needed for future research. Therefore, the weakness of this study was that the questions we used are difficult to compare with other studies, the strength of this study was that we collected more prevalence data.

### 4.4 Age patterns

Another important issue is the age pattern of awake bruxism and sleep bruxism, in other words, are these conditions increasing with age, stable over time, or self‐limiting conditions? Regarding sleep bruxism, research was performed in which the population was divided in several age groups in order to detect a pattern over time. Two studies reported a clear diminishing over time^4^, ^6^ (see Tables 4 and 5). Two studies showed first a stable period followed by a decrease^7^, ^9^ (see Table 4), in our study, a similar pattern was founded although our prevalence percentages are higher in all age groups. In a study assessing bruxism (without separating awake bruxism and sleep bruxism), a stable prevalence was shown as well^20^ (see Table 4), although the percentages are higher than in the previous discussed studies, most probably because of the summation effect. Regarding awake bruxism, to our knowledge, only one study reported age groups, showing stable prevalence percentages, with a peak in the age group 45‐54^4^ (see Table 5). In our study, we found also a stable prevalence, with the highest percentage in the first two age groups, followed by a decrease in the third and fourth age group, with a decline in the oldest age group.

**Table 4:** Comparison of different prevalence studies regarding “possible” sleep bruxism, assessing age patterns

	6. Lavigne & Montplaisir (1994)	7. Ohayon et al (2011)	9. Khoury et al (2016)	20. Ciancaglini et al (2001)
Type	SB	SB	SB	B
Age	↓Age	→↓Age	→↓Age	→Age
Gender	Male = female	Male = female	Male = female	Male = female
n	2019	13 057	6357	483
Age groups	4 see below (%)	5 see below (%)	6 see below (%)	5 see below (%)
		15‐18 (2.2)		
	18‐29 (13.0)	19‐24 (5.8)	18‐24 (22.0)	
			25‐34 (7.1)	<30 (34.6)
	30‐44 (9.0)	25‐44 (5.8)	35‐44 (10.9)	31‐40 (33.8)
	45‐59 (7.0)	45‐64 (4.7)	45‐54 (7.7)	41‐50 (29.5)
			55‐64 (5.5)	51‐60 (29.4)
	≥ 60 (3.0)	≥ 65 (1.9)	≥ 65 (4.4)	>60 (26.9)
	overall (8.0)	(8.2)	(8.6)	(31.4)

**Table 5:** Comparison of different prevalence studies regarding both “possible” awake bruxism and “possible” sleep bruxism, assessing overall rates or overall rates and age patterns

	4. Agerberg & Carlsson (1972)	4. Agerberg & Carlsson (1972)	5. Jensen et al (1993)	5. Jensen et al (1993)	8. Winocur et al (2011)	8. Winocur et al (2011)	Wetselaar et al (2019)	Wetselaar et al (2019)
Type	AB	SB	AB	SB	AB	SB	AB	SB
Age	→Age	↓Age	↓Age	↓Age	NM	NM	→↓Age	→↓Age
Gender	Female = male	Female = male	Female > male	Female > male	NM	NM	Female > male	Female > male
Age groups	5 see below	5 see below	1 25‐64	1 25‐64	1 18‐70	1 18‐70	5 see below	5 see below
n	1106	1106	975	975	402	402	1209	1209
25‐34	19.0	15.0					6.5	20.0
35‐44	20.0	9.0					7.8	21.0
45‐54	29.0	5.0					4.0	16.5
55‐64	24.0	4.0					3.2	14.5
65‐74	19.0	2.0					3.0	8.3
overall	20.0	10.0	22.1	15.3	31.0	14.0	5.0	16.5

NM, Not Mentioned.

Three studies used the same age groups, namely Agerberg & Carlsson,^4^ Khoury et al,^9^ and in our study as well, meaning 25‐34, 35‐44, 45,54, 55‐64 and 65‐74. Also, here consensus should be reached to compare future prevalence studies amongst each other.

Remarkable, when comparing prevalence studies were both sleep bruxism and awake bruxism were assessed, the three studies in Sweden, Denmark and Israel all founded a higher overall prevalence of awake bruxism compared to sleep bruxism^4^, ^5^, ^8^ (see Table 5). One can say that, on average, awake bruxism percentages are twice as high as sleep bruxism percentages. We found not only the opposite, meaning lower awake bruxism percentages than sleep bruxism percentages, but three times less, namely 5% towards 16.5%. In our opinion, the only explanation for this can be the fact that the exact question is different. We cannot think of other reasons why lower prevalence rates are assessed in the Netherlands as compared to Sweden, Denmark and Israel^4^, ^5^, ^8^ (see Table 5). The strength of this study was that we divided in age groups in order to detect patterns, we followed the most followed division in age groups so far.

### 4.5 Gender

With reference to gender differences, regarding sleep bruxism, the majority of studies reported equal prevalence for men and women,^4^, ^6^, ^7^, ^9^ that was also the case in the study regarding bruxism in general^20^ (see Tables 4 and 5). One study found a higher prevalence amongst women, this was the case both for awake bruxism and sleep bruxism^5^ (see Table 5). Also, in our study, both awake bruxism and sleep bruxism were reported more frequently by women than by men (see Table 2). This difference is statistically significant for awake bruxism only (*P* = 0.01).

### 4.6 SES

Since our study was part of a large epidemiologic survey on oral health of the general Dutch adult population, also the SES was assessed. It was revealed that awake bruxism and sleep bruxism were reported more frequently by participant with high SES (see Table 3). This difference is statistically significant for awake bruxism only (*P* < 0.05). In the other prevalence studies, Agerberg and Carlsson determined the level of education,^4^ Jensen and coworkers determined the sociodemographic variables,^5^ and Khoury and coworkers determined the SES, namely the years of education,^9^ but neither of them reported regarding the prevalence of awake bruxism and/or sleep bruxism and the SES.

### 4.7 Sample and sample size

Regarding the sample of the prevalence studies, all the authors could explain that the chosen target group is representative for the adult population. The Swedish study included 1.106 participants^4^ (Table 5), the Danish study included 975 participants^5^ (Table 5),and the Canadian study included 2.019 participants^6^ (Table 4). In the European study, a total of 13 057 participants were included (in the United Kingdom, 4972 in Germany, 4115 subjects respectively)^7^ (Table 4). Furthermore, in the Israelian study 402 participants were included^8^ (Table 5), in the Canadian study 6.357 participants^9^ (Table 4), and in the Italian study 483 participants^20^ (Table 4). It was mentioned in the Methods section that all edentulous participants were excluded from the analyses so only data of the dentate participants were analysed (n = 1209), this was done to make a comparison possible with the other prevalence studies. The selection procedure of our study is described in the Methods section. In addition, it can be reported that because of the fact that bruxism appeared to have a socioeconomic gradient and we have found that there was a slight difference in SES between respondents and non‐respondents, we have weighted the outcomes using the socioeconomic status of the different age groups. These weight factors were retrieved of the Health survey of Statistics Netherlands.^21^ Repeating the analyses using the weighted data did not result in statistically significant different results: Percentage of self‐reported sleep bruxism decreased by 0.4% to 16.2% and by 0.1% to 4.9% for awake bruxism. It was concluded that the used sample of 1209 participants can be regarded as representative for the Dutch adult population, which is a strength of this study. Also here, agreement is necessary, how to select representative samples amongst general adult populations.

### 4.8 Future perspectives

Awake bruxism and sleep bruxism are considered as two separate normal behaviours. There is a need to collect prevalence data for these conditions separately, in representative populations, that can be used for constructing a framework of “normality” (with respect to gender and age patterns). Consensus should be achieved regarding the exact questioning, since the questions itself can influence the results.

## 5 CONCLUSION

The results of assessing possible awake bruxism and possible sleep bruxism, being part of a large epidemiologic survey on oral health of the general Dutch adult population, revealed a prevalence of 5.0% of the total population for awake bruxism and 16.5% for sleep bruxism. Women reported to have both awake bruxism and sleep bruxism more often, these differences were statistically significant. Concerning socioeconomic status (SES), both awake bruxism and sleep bruxism were more often found in high SES groups, being statistically significant for awake bruxism only. Therefore, it was concluded that sleep bruxism is a common condition in the Dutch adult population while awake bruxism is seen more seldom.

## CONFLICT OF INTEREST

The authors declare no conflicts of interest.
